# Supplementary material for: GDF-15 Deficiency Reduces Autophagic Activity in Human Macrophages In Vitro and Decreases p62-Accumulation in Atherosclerotic Lesions in Mice
Source: Cells. 2021 Sep 7;10(9):2346. doi: 10.3390/cells10092346 (PMC8470202; doi:10.3390/cells10092346)
Supplement: Supplementary file 1 [file cells-10-02346-s001.zip › cells-1258020-supplementary.pdf]

Table S1. Used antibodies for western blot.

Antibody	Description	Cat. NO	Producer	Dilution	MW (kDa)
Primary Antibodies					
anti-alpha tubulin	rabbit anti-alpha tubulin antibody	Ab4074	Abcam plc., Cambridge, UK	1:4000	50
anti-LC3A/B	rabbit anti-LC3A/B antibody	#12741	Cell signaling	1:1000	14/16
Secondary Antibodies					
anti-rabbit IgG	donkey anti-rabbit IgG, HRP-linked F(ab')$_2$-fragment	NA9340	GE Healthcare Life Science Freiburg, Germany	1:6000	

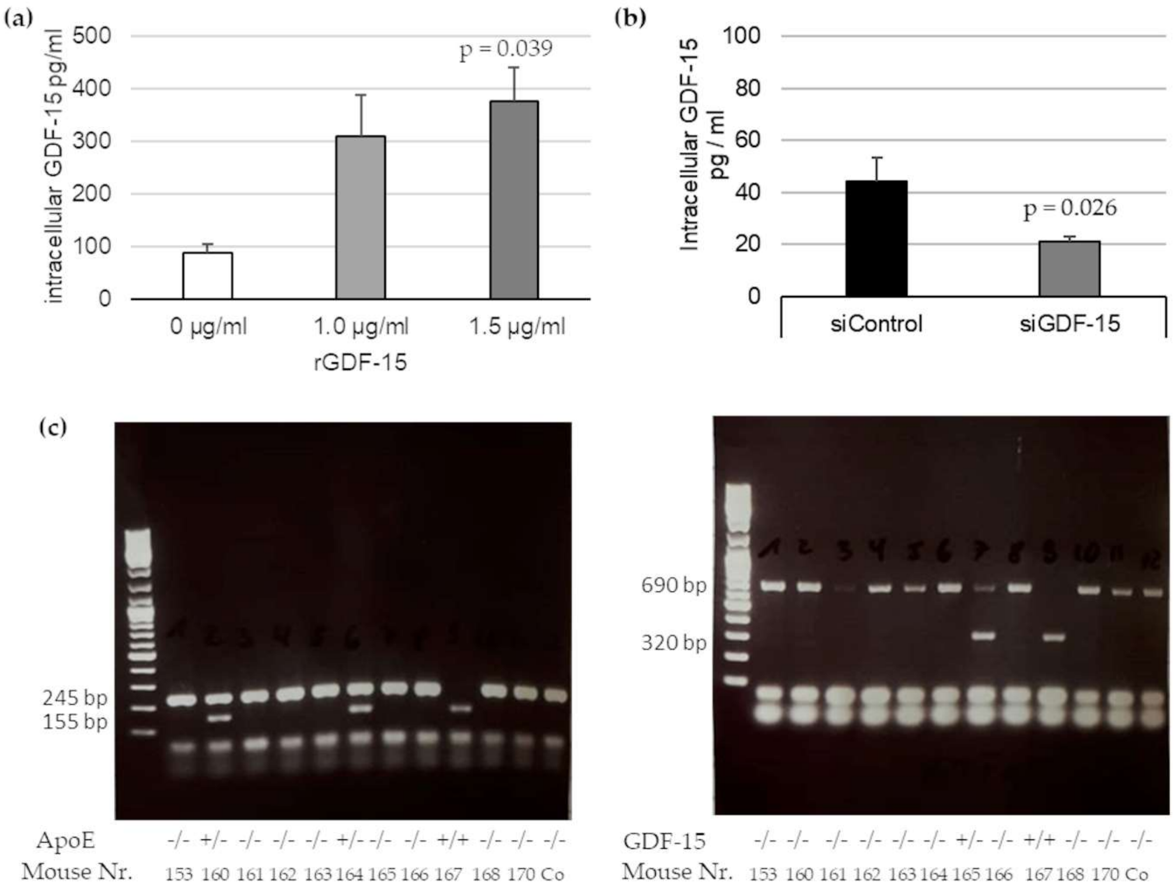

Figure S1. GDF-15 protein level in human THP-1 MΦ and genotyping of GDF-15$^{-/-}$ / ApoE$^{-/-}$ mice. **(a, b)** Intracellular GDF-15 level (in pg/ml) of THP-1 MΦ were determined using ELISA [$OD_{490/655}$] (three independent experiments were performed). **(c)** PCR used for genotyping GDF-15$^{-/-}$ / ApoE$^{-/-}$ mouse lines. Mouse ear DNA was amplified with primers previously published [18]. -/- = knockout; +/+ = wild type; +/- = heterozygote; Co = positive control
